# Prediction methods for microRNA targets in bilaterian animals: Toward a better understanding by biologists

**DOI:** 10.1016/j.csbj.2021.10.025

**Published:** 2021-10-18

**Authors:** Aurélien Quillet, Youssef Anouar, Thierry Lecroq, Christophe Dubessy

**Affiliations:** a Normandie Université, UNIROUEN, INSERM, Laboratoire Différenciation et Communication Neuronale et Neuroendocrine, 76000 Rouen, France; b Normandie Université, UNIROUEN, UNIHAVRE, INSA Rouen, Laboratoire d'Informatique du Traitement de l'Information et des Systèmes, 76000 Rouen, France; c Normandie Université, UNIROUEN, INSERM, PRIMACEN, 76000 Rouen, France

**Keywords:** MicroRNA, Target prediction, Bioinformatics tools, Computational prediction methods, Data combination, Performance evaluation

## Abstract

MicroRNAs (miRNAs) are small noncoding RNAs that regulate gene expression at the posttranscriptional level. Because of their wide network of interactions, miRNAs have become the focus of many studies over the past decade, particularly in animal species. To streamline the number of potential wet lab experiments, the use of miRNA target prediction tools is currently the first step undertaken. However, the predictions made may vary considerably depending on the tool used, which is mostly due to the complex and still not fully understood mechanism of action of miRNAs. The discrepancies complicate the choice of the tool for miRNA target prediction. To provide a comprehensive view of this issue, we highlight in this review the main characteristics of miRNA-target interactions in bilaterian animals, describe the prediction models currently used, and provide some insights for the evaluation of predictor performance.

## Graphical abstract

**Figure:**
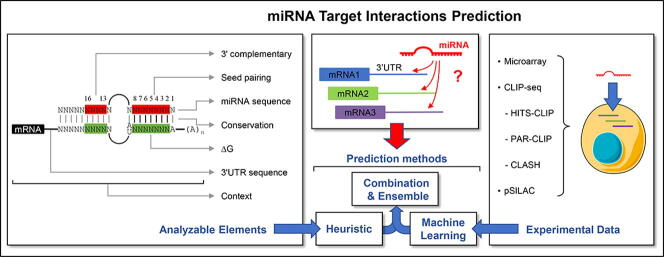

## 1 Introduction

MicroRNAs (miRNAs) are small (∼22 nucleotides) noncoding RNAs that act as posttranscriptional regulators of gene expression for all known biological processes [1]. Indeed, between 60 and 90% of human genes are believed to be regulated by miRNAs, as revealed by genome-wide analyses [2], [3]. According to miRbase (release 22), the primary database of published miRNA sequences and their annotation, a total of 48,885 mature miRNA products have been identified in 271 eukaryotic species, among which 2654 are found in humans [4]. Animal and plant miRNAs share many similarities in their biogenesis and mode of action, as revealed by biochemical and genetic studies, suggesting that they share a common ancestral origin. However, the sites of biogenesis of miRNAs, their genetic structure or the location of their genes differ between plants and animals [5], [6], [7], [8].

In bilaterian animals, miRNAs are mostly transcribed by RNA polymerase II, which yields a primary miRNA (pri-miR). This pri-miR is then processed to generate a miRNA precursor (pre-miR) by the microprocessor complex composed of DROSHA and DGCR8 (known as Pasha in flies and nematodes). Then, exportin-5 and RAN-GTP transfer the pre-miR from the nucleus to the cytoplasm to be further processed by DICER and produce the mature-miRNA duplex sequence. The biogenesis of miRNAs in animals has been reviewed in several publications [1], [9], [10], [11], [12], [13], [14]. The miRNA inhibition process requires the formation of miRNA-induced silencing complexes (miRISCs), which are mainly composed of the Argonaute (AGO) family of proteins and several other proteins, such as the trinucleotide repeat containing 6 (TNRC6, known as GW182 in flies) family of proteins [14], [15], [16]. These proteins are mainly localized in cytoplasmic P-bodies, which are considered the primary sites of miRNA activity in the cytoplasm, although they can also occur in many cellular compartments, such as the nucleus, mitochondria or vesicles of the endosomal trafficking pathway [17]. In most cases, miRISC induces silencing through a combination of processes, including translational repression, deadenylation, decapping and 5′-to-3′ mRNA degradation [18], [19]; however, mRNA decay is believed to be responsible for 66–90% of silencing [20], [21]. Interestingly, plant miRNAs regulate their targets mainly by binding with nearly full complementarity to unique sites in the coding region. This high pairing rate mostly leads to endonucleolytic mRNA cleavage and a strong effect on a limited number of targets [6]. In contrast, miRNAs from bilaterian animals regulate transcripts via imperfect complementarity at multiple interaction sites mainly located in the 3′-UTR, which allows them to potentially regulate several hundred mRNAs, and one mRNA can be targeted by several miRNAs [13], [22], [23], [24], [25]. Because of these numerous possible interactions, miRNAs exert major effects in a variety of cellular processes, including cell proliferation, migration, apoptosis and differentiation [26], [27], [28]. Consequently, altered expression of miRNAs has been observed in many pathologies [29], including cardiovascular [30], neurodegenerative [31], and renal diseases [32], and most notably in cancers [33], [34], [35]. Therefore, improved knowledge of the mechanisms of action of miRNAs will likely impact our understanding and management of these diseases.

Because miRNAs are now considered major actors among noncoding RNAs for the regulation of gene expression, their role in this important cellular mechanism has been an expanding area of research since 2001. This is particularly challenging in bilaterian animals due to the imperfect interaction between miRNAs and their target mRNAs and the resulting large number of potential targets. To understand this role, it is essential to identify functional miRNA targets in a predefined cellular and environmental context. This goal could be achieved through the use of a combination of cell biology techniques, including gene reporter assays, quantitative PCR and western blot [36]. While a Luciferase gene reporter test can identify the direct interaction between a miRNA and its targeted mRNA region, qPCR and western blot assess the transcriptional and translational repression resulting from the interaction [36], [37]. These techniques are time-consuming and allow validation of a few interactions at a time. To address this issue, cross-linking and immunoprecipitation approaches coupled with next-generation sequencing (CLIP-seq) have been developed. These techniques allow massive discovery of miRNA target interactions (MTIs) without the need for miRNA overexpression. However, the identified interactions still need additional investigations to decipher their biological meaning [36], [38]. Although improvements have been made, many datasets generated by this approach contain numerous false-positives due to UV crosslinking issues [39]. Regardless of the experimental procedures, they are time-consuming and expensive; thus, in silico MTI predictions are required. Predictions of novel target sites could be achieved by building a classification or ranking model based on experimentally validated MTI properties (further described below). During the last decade, scientists have proposed many different computational approaches, although a consensus has not been reached on how to best predict MTIs. Currently, more than 192 target prediction tools have been described (as of November 2020, from OMICtools’ database) [40]; therefore, it is difficult to find the best suited tool for the analysis of a particular experiment. This issue has been the subject of several reviews that discuss common prediction tools as well as the main characteristics of MTIs [41], [42], [43], [44]. Recently, Kern et al. proposed a dedicated tool that would facilitate the choice of the most appropriate prediction tool [45]. However, computational predictions present high false-positive/negative rates due to the small size and the binding complexity of the MTI sites [46]. Moreover, without a common method to evaluate them, it is not easy to decide which one to test first. Indeed, the result lists given by each MTI prediction algorithm for a given miRNA differ greatly in the targets identified, prediction number and ranking [47]. Below, we will describe the main characteristics of MTIs in bilaterian animals as well as different up-to-date computational methods that could help biologists choose the appropriate tool, and we will provide the knowledge necessary to avoid the numerous drawbacks of these prediction tools. The issue of algorithm performance evaluation will also be addressed.

### 2 Analyzable elements

Although the mechanisms of action of miRNAs are not fully understood, several features of MTI have been defined through experimental work. Although each algorithm uses a different set of features, sequence complementarity, site accessibility and sequence conservation are the most commonly used.

#### 2.1 Sequence features

##### 2.1.1 Seed region

**Fig. 1:**
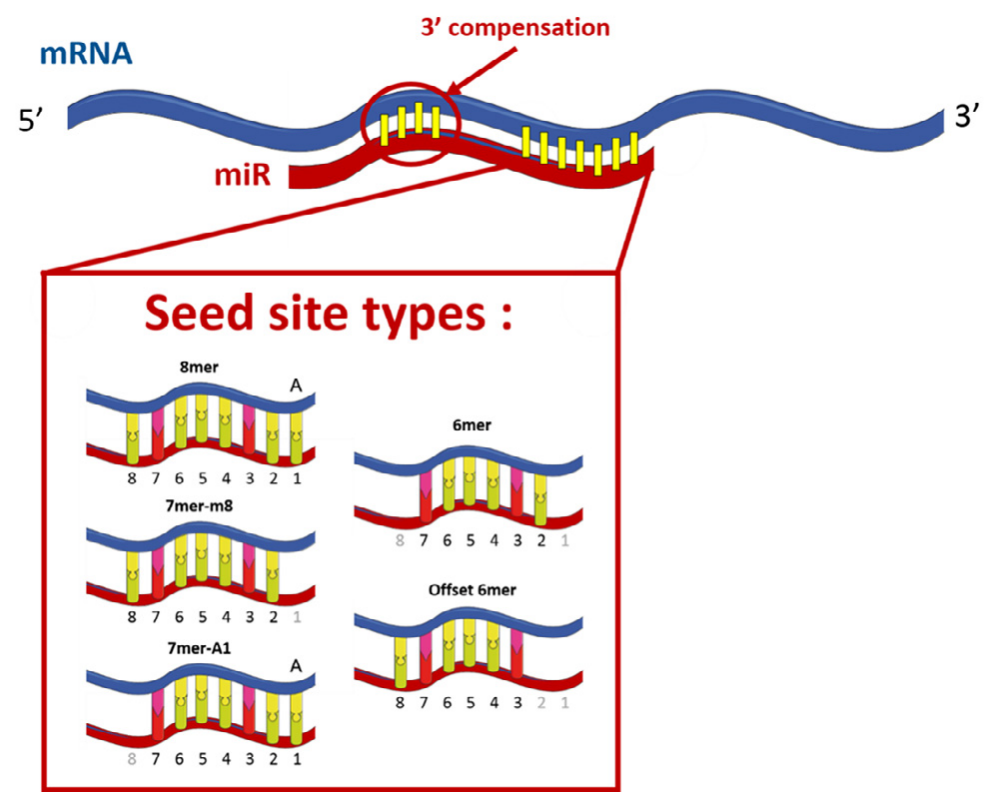
microRNA seed site types. The vast majority of miRNA interactions occur through several matching possibilities of the seed region as described above. Mismatches in the seed region can still result in a functional interaction with the help of 3′ compensatory pairing.

The main biological feature underlying the interaction between miRNA and mRNA is defined as the “seed” region, which includes nucleotides (nt) 2 to 8 starting from the 5’ end of a miRNA. A perfect match with the seed region does not always induce mRNA repression, clearly indicating that this parameter alone is not sufficient to predict the interaction [48], [49], [50]. Interestingly, the recognition of an adenine at miRNA nt 1 favors miRNA-mediated protein downregulation even when it does not participate in a Watson-Crick interaction [51]. Seed sites are categorized into different types according to their pairing degree. The hierarchy of site efficacy is as follows: 8mer ≫ 7mer-m8 greater than 7mer-A1 ≫ 6mer or offset-6mer (position 3–8 match) > no site, with the 6mer differing only slightly from no site at all (Fig. 1) [2], [50]. Microarray experiments suggest that the majority of miRNA target sites are 7mer-m8 type [50]. The difficulty of using the seed region in target prediction is based on the occurrence of “bulges” (unpaired stretches of nucleotides located in either one of the sequences) or G:U wobbles within the sequence that reduce (but do not prevent) inhibition efficiency [48], [51]. These sites are named “orphans” or “noncanonical” because AGO proteins can bind them without a perfect seed match. They were thought to be relatively rare in mammals [2], [52], [53], [54]. However, more recent experimental methods tend to identify a much higher number of noncanonical sites or even sites not binding to the seed region at all (binding to the center of the miRNA or 3’ end) [51], [52], [55], [56], [57], [58]. A possible explanation for some of these noncanonical sites is the existence of a “pivot bulge” on the 6th nt of the seed that could enable a transitional nucleation state by stabilizing nucleation base pairing (positions 2–6), allowing subsequent bulge formation and propagation of the seed interaction [53], [59]. An alternative hypothesis is that noncanonical sites, since they are poorly conserved across species, may act as evolutionary intermediates between nonfunctional sites and canonical target sites with selection pressure going toward the appearance of higher affinity sites [54]. In any case, functional assays indicate a mild regulatory effect of these noncanonical sites [50], [53], [57]. Therefore, the usefulness of considering both fully and partially matching seed sites to improve MTI prediction is still a matter of debate [60], [61].

##### 2.1.2 Compensation

While most studies consider a “canonical” site to be a full seed pairing without a bulge, miRNA target sites can in fact be divided into three groups: canonical (or seed only), atypical canonical and noncanonical sites [14]. Canonical sites, which were described in the previous paragraph, have strong 5’ pairing but require little or no 3’ pairing. Atypical canonical sites have both strong seed pairing and supplementary pairing on the 3’ side of the miRNA. Finally, noncanonical sites have weak seed pairing and strong 3’ pairing. One might think that atypical canonical sites are more effective than seed sites only. However, evaluating the effectiveness of 3’ supplementary pairing is very difficult due to the number of pairing possibilities and the dependence on context of this parameter [14]. Nevertheless, additional Watson-Crick pairings of at least 4 nt at positions 12–17, especially from 13 to 16, enhance miRNA targeting [50]. This type of strong compensation is very rare (less than 2% of known conserved MTIs), although when it exists, its target site is usually highly conserved across species [2].

##### 2.2 Site accessibility

The complexity of miRNA-mRNA interactions leads to the rather weak efficiency of algorithms based on sequence matching only. Additional parameters, such as thermodynamics, UTR context or site conservation, must be considered. Site accessibility is as important as individual nucleotide matches in the seed since the action of a miRNA is mediated by a relatively large silencing complex.

###### 2.2.1 Thermodynamic effect

The most basic approach for considering the thermodynamic effect is to calculate the free energy, which reflects the stability of the RNA binding sequences. This binding is believed to form a stable, low-energy duplex. Therefore, lower energy values indicate a more plausible interaction. Since we are in the context of miRNA interactions, constraints imposed by seed pairing must be taken into consideration. The ViennaRNA R package is the most commonly used tool to calculate the free energy of binding. It aggregates more than 20 programs/packages to solve the structure of an RNA duplex using dynamic programming [62]. Rehmsmeier *et al.* found that forbidding intramolecular base pairing and bulge loops seems to give a better free energy estimation [63]. They also noted that taking several nt (10 and more) flanking the target site improves the correlation between energy-based scores and target repression [63], [64]. Another possibility is to consider the hybridization energy (ΔΔG), which is the difference between the free energy gained by the binding of the miRNA to the target, ΔG_duplex_, and the free energy lost by unpairing the target-site nucleotides, ΔG_open_. This ΔΔG score correlates well with the degree of miRNA target repression for some interactions but not all [64].

###### 2.2.2 Target site context

Messenger RNAs can fold into highly elaborated secondary and tertiary structures, and a perfect miRNA sequence match might not be structurally accessible for binding. Therefore, contextual features, such as the local AU nucleotide composition, proximity to residues that can pair to miRNA nucleotides 13–16, or positioning away from the center of long UTRs, must be included in MTI prediction algorithms. Among all contextual features, the AU content around the target site favors most of the interactions with a miRNA [22]. Indeed, swapping a target site from an open (AU rich) UTR structure to a close structure decreases the site functions [64]. A possible explanation for this phenomenon is that AU-rich sequences could be recognized directly by a RISC component or may reduce the tendency to form stable RNA secondary structures that could interfere with RISC binding [65]. Although there is a high prevalence of MTI sites found in the 3′-UTR, some miRNAs can also regulate mRNAs by binding to the 5′-UTR and the coding sequence (CDS) region of their targets [66], [67]. However, target sites in the open reading frame are not as efficient as the other sites [51], [68], [69]. Interestingly, a recent study showed that the sites in the CDS are quite potent at inhibiting translation by inducing transient ribosome stalling instead of mRNA destabilization [70], [71]. Interestingly, some studies have shown that miRNA interactions with different binding sites and/or under different cellular conditions can increase mRNA translation [72], [73], [74]. However, the precise mechanism by which a miRNA can enhance protein synthesis remains to be elucidated. Thus, it is important not to restrict the search for MTI predictions to the 3′-UTR. Aside from localization, the number of repetitions of a target site and their spacing on a given mRNA also affect the inhibition efficiency of a miRNA [65], [75]. Another important aspect to determine the possibility of an interaction, yet rarely taken into consideration, is the expression level of both miRNAs and targeted mRNAs [76]. Moreover, depending on the tissue or disease, a validated MTI can be more or less functional [77], [78]. This might be due to RNA-binding proteins that could block access to miRNA or mRNA secondary structures in that particular tissue or disease [78], [79]. Conversely, certain RNA-binding proteins, such as PUM1 and Sfpq, have been shown to promote miRNA targeting [79], [80]. These RNA-binding proteins in each tissue or disease of interest must be considered to improve the predictions of MTIs.

##### 2.3 Conservation

The level of conservation of a sequence corresponds to its presence across different species. The use of the evolutionary conservation of miRNA targets is motivated by the idea that closely related species should share common MTI sites. However, most target sites are not fully conserved over their entire length, with higher conservation often occurring in the seed region than in the other sequences of the target site. Moreover, only the percentage of 3′-pairing is generally conserved and not the nucleotides themselves. Assuming that aligned sites within orthologous genes have a common origin, it was proposed to quantify site conservation in a phylogenetic tree by summing the length of all branches in which the site is present [81].

Of note, the level of conservation of a target site has to be estimated with regard to the conservation of its mRNA region and its length [2]. A stronger conservation profile has been associated with increased mRNA downregulation as assessed by microarray experiments and better MTI prediction [2], [51], [65], [82], [83]. Indeed, over 60% of human protein-coding genes have conserved targets for miRNAs, thus supporting the importance of this parameter [2]. However, since functional nonconserved MTIs exist and mediate protein translation inhibition [84], target sites cannot be filtered based on conservation criteria only. Agarwal *et al.* also observed a decrease in the performance of their predictor when considering only highly conserved sites [60]. Therefore, an ideal equilibrium must be found where conserved sites are favored as well as where nonconserved sites are also retained. Friedman *et al.* reported a high number of preferentially conserved 6mer sites [2], a surprising finding since, as mentioned above, 6mer sites typically have poor efficacy when examined experimentally [50]. A possible explanation for this result is that these sites are inactive (or less active) forms of conserved 7–8mer sites. An alternative explanation is that when binding with a 6mer, miRNA induces a function other than repressing protein output. For example, a role in mRNA subcellular localization could allow many 6mer sites to be conserved while having a poor effect on protein level inhibition [2].

### 3 Computational prediction methods

As mentioned in the introductive part of this review, many computational tools have been developed in the field of MTI prediction. The main objective of prediction algorithms is to select the most discriminative features within the categories of analyzable elements described above and to determine the best way to compute them to obtain the most accurate prediction.

##### 3.1 Sequence based

###### 3.1.1 Heuristic scoring models

The earliest attempt to identify miRNA targets in silico was published by Stark *et al.* in 2003 [85]. The screening performed in this study was a simple two-step procedure combining sequence comparison with HMMer (alignment tool) and site accessibility using Mfold. The resulting targeted 3′-UTRs were then compared based on their conservation between *Drosophila pseudoobscura* and *Anopheles gambiae*, and they successfully validated 6 MTIs for two *Drosophila* miRNAs that they predicted using this protocol. After analyzing the characteristics of these 6 validated interactions, they described what we now know as the seed region: nucleotides 2 to 7 at the 5′ end of miRNA [85].

Following this initial report, many more studies have been performed with the aim of improving and generalizing MTI prediction. The vast majority of the described predictors utilize the seed-matching parameter since most of the reported functional MTIs have a 6mer or more. To determine this parameter, predictors either filter sequences based on a defined set of rules for seed matching [60], [86] or use a score system that favors this feature [24], [87], [88]. However, filtering based on seed rules seems too stringent because functional MTIs can also have noncanonical seed sequences (G:U wobble or bulge). In this regard, some methods consider the binding of the first eight nucleotides as important but do not restrict it to particular seed types [89], [90], [91], [92]. MIRZA-G (evolution of MIRZA [93]), for instance, is a recently published algorithm that allows for nonperfect seed matches if the final score for the site is above the author-defined threshold [83]. Predictors such as RNA22 [3] that do not consider seed matching at all in their predictions are rather rare. In the case of RNA22, the algorithm probes mRNA for patterns generated by comparing all known mature miRNA sequences (as of 2006) and keeps only the most similar ones. Sequence alignment results are almost always complemented with site accessibility and evolutionary inputs. Tools such as miRanda [88], RNA22 [3] and TargetScan [60], [94] make use of RNA folding prediction software, such as RNAVienna [62]or Mfold [95] packages, to estimate the free energy of predicted miRNA–target duplexes and filter out the candidates above a certain threshold. Interestingly, the authors of RNAhybrid [63] used a different approach that avoids intramolecular base pairing and bulge loops, which seems to improve the estimation of the free energy [63]. In fact, some predictors, such as PicTar [24] and STarMir [96], [97], use the results of RNAhybrid to filter potential target sites. As mentioned before, the authors of other predictors, such as PITA [64], prefer to consider the hybridization energy (see “Thermodynamic”: II.B.1) to score miRNA–target duplex stability. Out of all the site accessibility features, the local AU content is the most implemented since it has been shown to favor MTI [60], [89], [90], [92], [98], [99]. The frequency of target sites along the mRNA and the distance separating them are two other features often considered for target site context implementation [21], [94], [100]. The value of site conservation is frequently advocated since omitting nonconserved targets and not using this parameter drastically decrease the specificity of the method [2], [90], [94], [101]. This parameter has been extensively analyzed by the authors of EIMMo [86], who scored MTIs based on conservation criteria only and then used Bayesian statistics to infer functionality. Therefore, EIMMo is quite efficient at predicting the mRNAs targeted by a given miRNA but not as sensitive at the target site level [102]. Feature implementation for all the algorithms cited thus far has been performed based on literature data only. To better identify the combination of features to use, the authors of miRmap decided to evaluate each of them individually before integrating them. They first screened all human transcripts for 7mer seeds and compared the performance of eleven features mentioned previously on the results from seven miRNA overexpression experiments obtained in five different studies. Based on this evaluation, they combined these features using a linear regression model, thus making it the most comprehensive MTI predictor at that time [98]. Similarly, TargetScan evaluates 26 features and eventually selects 14 to upgrade itself using a similar model in 2015 [60]. Most algorithms store the identified interactions in a publicly available database format, such as miRWalk2.0 [60], [103].

###### 3.1.2 Empirical machine learning models

The limit of rule-based methods comes from the complexity of MTIs. It is extremely difficult to take into consideration all possible aspects of these interactions. Thus, another promising direction toward better MTI prediction is data-driven (or machine learning, ML) algorithms. There are many computational models available to build such an algorithm. Unfortunately, there is no fixed rule to select one for a given problem. In general, ML methods are categorized into two groups depending on whether the output values are present in the training data (supervised learning) or not (unsupervised learning). In the field of MTI prediction, all data-driven methods use supervised learning regression (scoring system) or classifiers (categories) to differentiate functional from nonfunctional sites. The performance of each method depends on the amount and quality of the training data, the complexity of the relationship between the inputs and outputs, and the local computational restrictions (time and memory). Computational constraints depend mostly on the number of features used [104]. Since an ML approach can only be as effective as the dataset used to train it, a large high-quality dataset is therefore primordial to build an accurate model. An ideal experimental dataset would contain all types of functional MTI and as many negative experimental examples, and it would also be free from any experimental biases. Since the precise mechanism of miRNA binding is not yet completely known, the aim of a data-driven algorithm is to find the best compromise of features to obtain a generalization model [105] capable of classifying an MTI in a binary fashion or according to a scoring method. Features are ranked by a metric system such as F-score (harmonic mean between precision and recall) or correlation coupled with statistics, and the top-ranked features are selected to build the algorithm. This procedure is known as feature extraction. To validate their approaches, most authors use a k-fold cross validation technique. In other words, a subset of the dataset is used for training the algorithm and the other part is used for testing it. This process is performed in general 10 times using different partitions of the original dataset, and the performance results are averaged over the rounds.

####### 3.1.2.1 Genetic programming

**Fig. 2:**
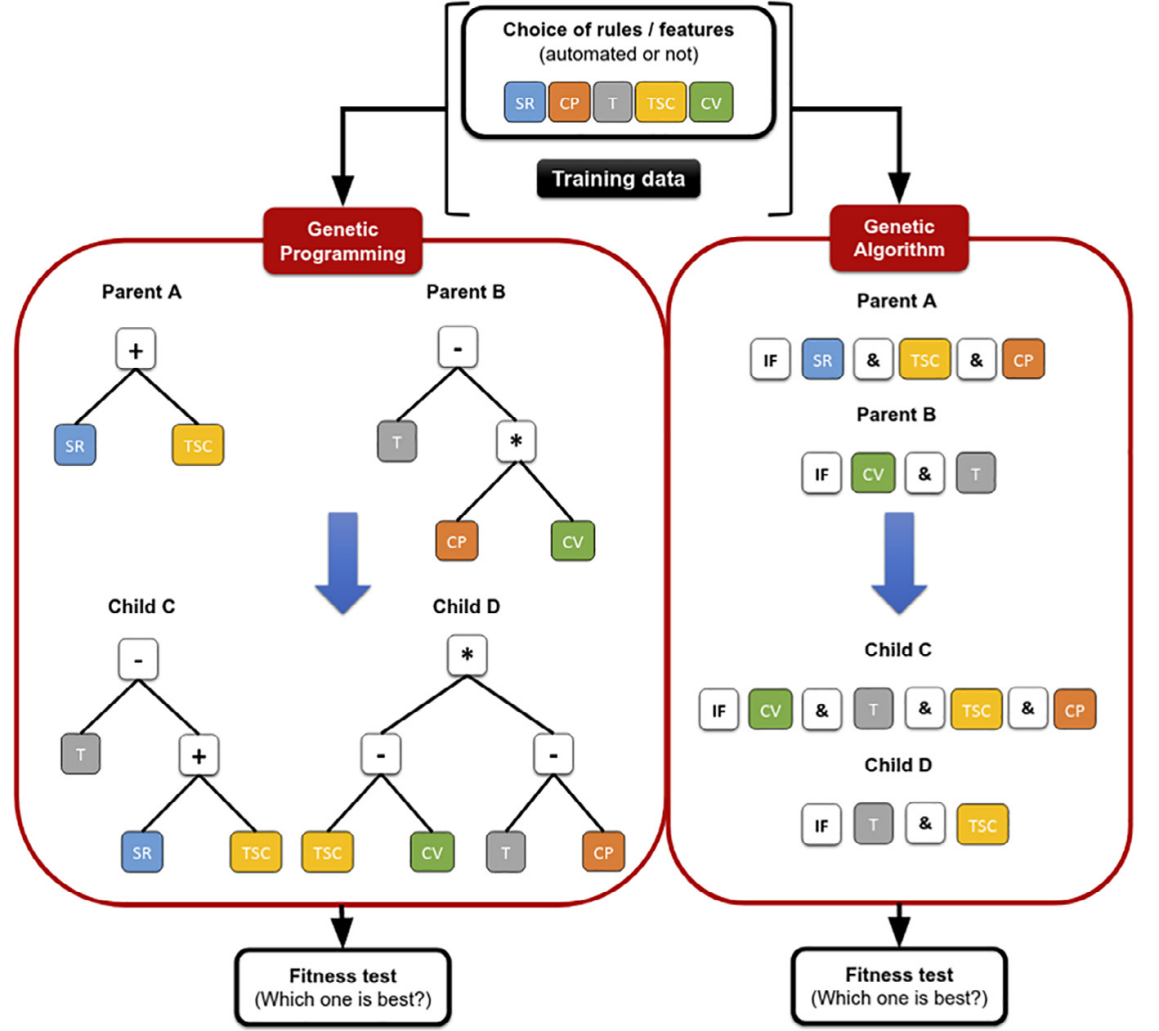
Basic schematics for the genetic programming (GP) and genetic algorithm (GA). Using seed region (SR) pairing, compensation (CP) pairing, thermodynamic (T) pairing, target site context (TSC) pairing and conservation (CV) pairing on the training data, both the GP and GA will create subtree crossovers of parents A and B to form offspring C and D. A fitness test is performed for each tree (parents and offspring) to decide which one is best suited for the classification of the training data.

Genetic programming is an ML method that generates functions (represented as trees) using the different rules or features implemented to best describe a positive interaction [106], [107] (Fig. 2). One of the first ML models developed with this method was TargetBoost in 2005 [107]. This model is one of the rare types of algorithms that does not use the seed matching criteria to predict MTIs. Instead, TargetBoost creates sequence motifs from a set of 36 experimentally validated MTIs (from the literature) and 3,000 random strings of 30 nt as negative examples. These motifs are then weighted with a boosting algorithm that eventually returns a score indicating the probability of interaction. Boosting algorithms combine a set of simple rules (or features) by assigning to each one of them a weight. The idea is to form a single model with better performance than each rule taken individually [106]. The final score is calculated by summing the number of true and false-positive/negative hits and the relative weights given by the algorithm for each sequence. Feature extraction is not performed, and conservation or site density filters are not applied in this model. The data from 3 miRNAs were used to train the model, which was tested on the data of another miRNA using the “leave one out” method. Compared with RNAhybrid and another algorithm named nucleus, TargetBoost was either as good as or more performant depending on the dataset used for testing [107]. To improve the performance of this type of model, a recent study by Rabiee-Ghahfarrokhi *et al.* used a genetic algorithm (Fig. 2) in combination with a C4.5 decision tree instead of boosting [108]. The output of the C4.5 algorithm results in several rule sets that can be taken as inputs for the genetic algorithm. First, their algorithm was trained and tested on a small dataset taken from the TarBase database (version 3.0) and containing 48 positive and 16 negative examples [109], [110], [111]. They obtained 94% accuracy using a 10-fold cross-validation method for testing. This performance was confirmed by training and testing the model on a different dataset (taken from Ahmadi *et al.* [112]) containing 113 positive and 312 negative examples and therefore showed 97% accuracy. The authors related the high performances of their method to the set of rules used as inputs. However, in both cases, the training and testing datasets were not independent, thus increasing the likelihood that this algorithm will perform well.

####### 3.1.2.2 Probabilistic based classifier

**Fig. 3:**
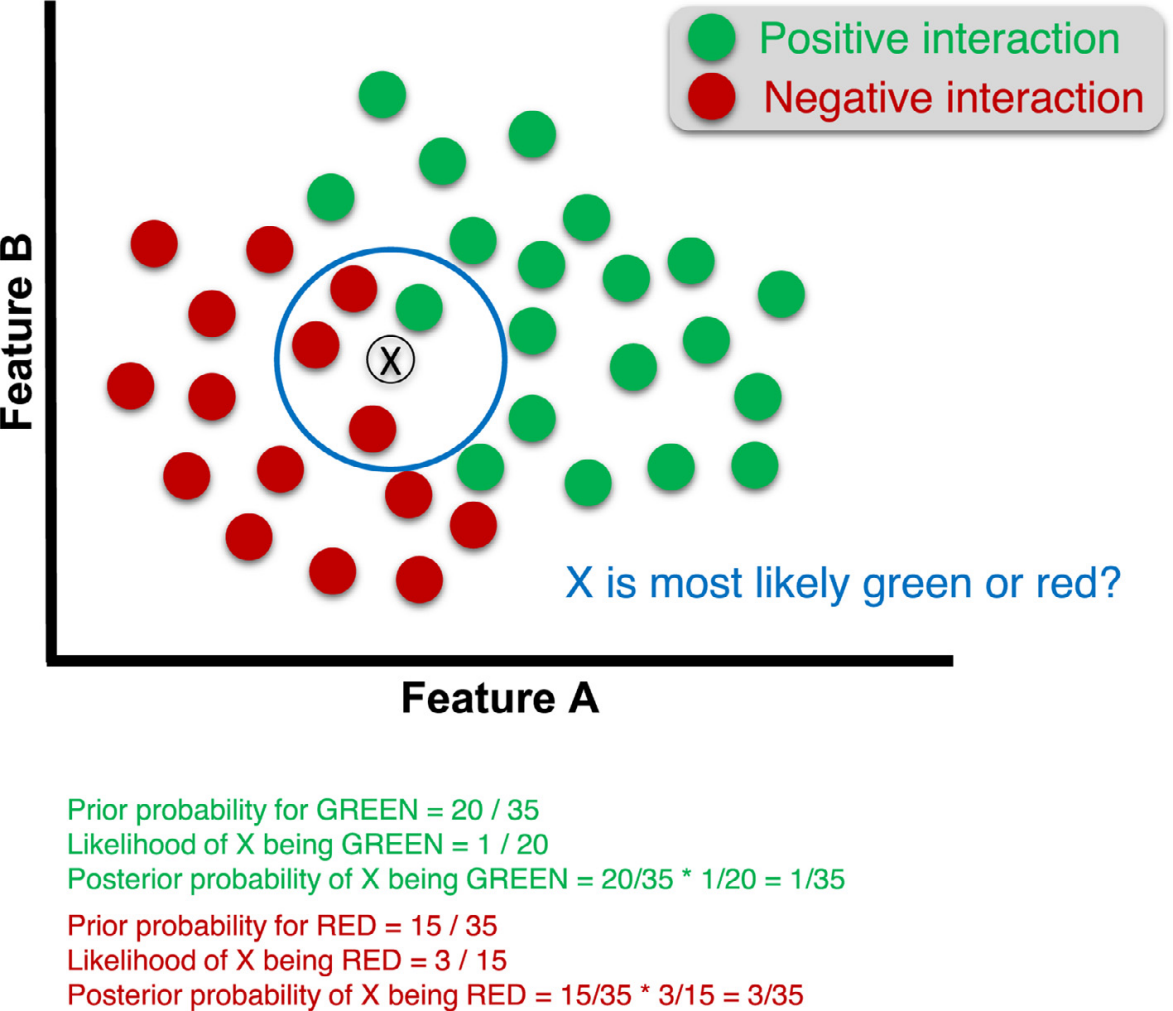
Naive Bayes classification. The probability that a given interaction is positive or negative is calculated for multiple sets of features. The final decision of the algorithm is the product of all probabilities.

A commonly used method is to model the relationship between the features and the output categories using probabilities with a naive Bayes (NB) classifier. In other words, this model computes the probability that a feature belongs to a certain class (in our case, positive or negative). An MTI is then classified based on the product of the probabilities of all features (Fig. 3) [104]. NBmiRTar is an example of such a probabilistic machine learning method [113]. Using both ‘seed’ and ‘out-seed’ features, the NB classifier was applied to predictions from miRanda, with its scoring and free energy calculation taken as filters. Moreover, the same dataset of 3000 random 30 nt strings were used as negative examples for the TargetBoost method. Interestingly, the two most important features in this model discriminate seed pairing mismatches (“number of bulges in the seed” and “number of bulges in the seed with length 1”). To avoid excluding nonconserved MTIs, the authors did not use sequence conservation in their model, which generates a large number of MTIs. Nevertheless, they claim to be able to reduce this number of MTIs while retaining most of the positive targets (10 out of 13) by using a high score threshold. However, the consistency of this model needs to be tested on more than 13 positive targets. Additionally, using a Bayesian probabilistic method, GenMiR3 [114] (an evolution of GenMiR++ [115]) considers the hybridization energy, target site conservation (PhastCons algorithm [116]) and context information (5 sequence features) to establish a prior probability for the target site to be functional. The authors tested the performance of each feature using multiple linear regression models and cross-validation and found that hybridization energy had the greatest enhancing effect on the predictive power of this model. Expression data for miRNAs and mRNAs were also used to compute a final (or posterior) probability for the site to be functional. Unfortunately, no performance evaluation is available for GenMiR3. Interestingly, although the training data was restricted to colorectal cancer MTIs, the CRCmiRTar authors compared different ML approaches (NB, SVM, random forest (RF), artificial neural network (ANN)) and found that the NB classifier was the most sensitive and specific method [117]. This algorithm also proved to be more efficient than other tools on an independent colorectal cancer-specific test dataset. The tissue origin of the samples therefore seems to be a parameter that should be included in MTI predictions.

**Fig. 4:**
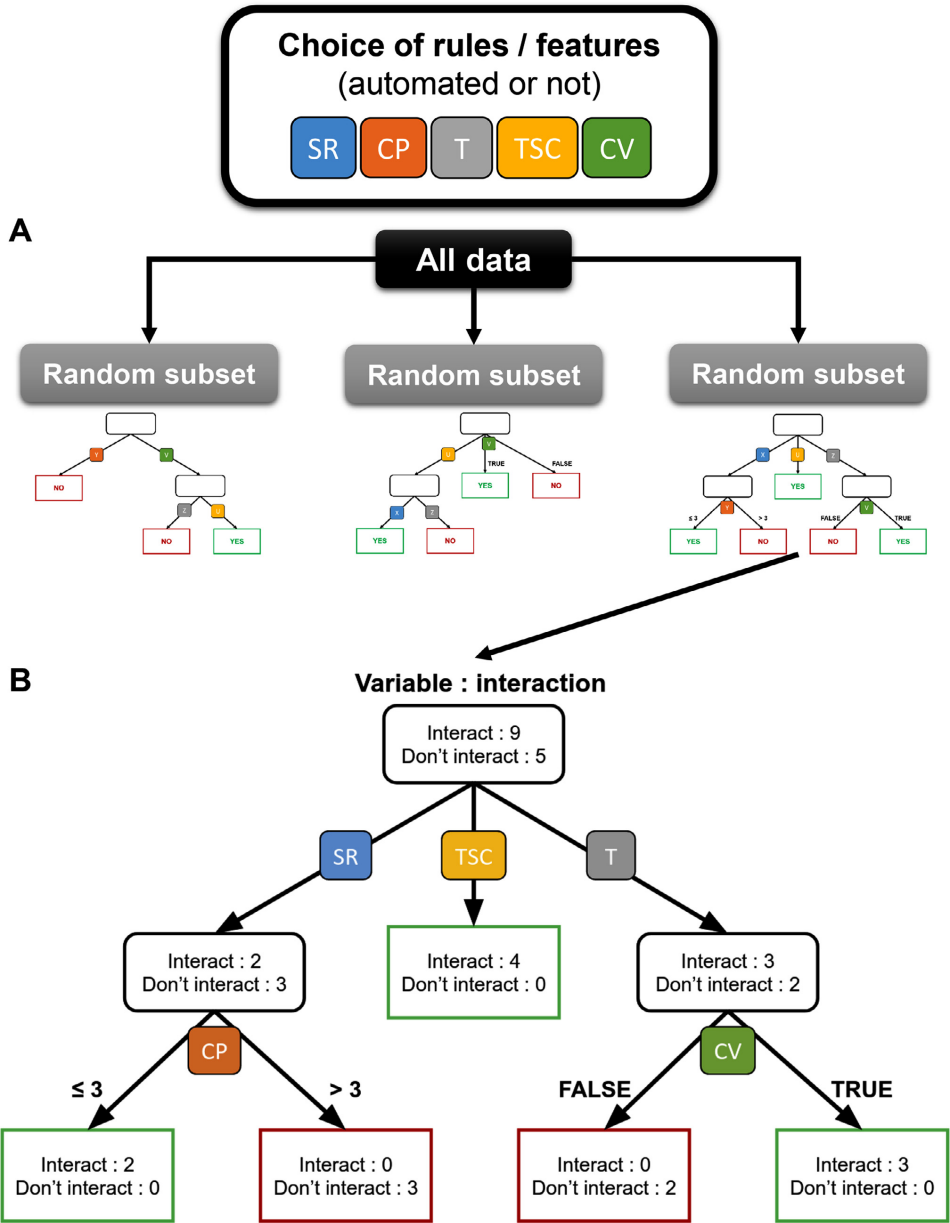
Random forest (RF) classifier. A) All data are randomly sorted into subsets to generate several trees using a predefined set of rules to optimize the split. In this example, seed region (SR) pairing, compensation (CP) pairing, thermodynamic (T) pairing, target site context (TSC) pairing and conservation (CV) pairing are used. B) This specific tree considers an interaction to occur if it possesses all necessary parameters to fall in one of the green leaves. The RF algorithm returns the prediction made by the majority of the trees. (For interpretation of the references to colour in this figure legend, the reader is referred to the web version of this article.)

Another probabilistic model used for MTI predictions is the random forest (RF) classifier. Each tree of the forest is a predictor that depends on the values and order of a randomly selected subset of features. When an unlabeled example is given to the algorithm, each tree votes, with the majority defining the predicted class for this example (Fig. 4) [118]. The mechanism used to grow the trees allows us to easily estimate the most important set of features and is also easily interpretable. An example of such a model is RFMirTarget [119]. The authors used the dataset published by Bandyopadhyay and Mitra [99] that contains 289 experimentally validated functional pairs and 289 “systematically identified tissue-specific negative examples” to train an RF classifier. Since no site alignment was given in this dataset, they used miRanda to define potential MTI site sequences and alignments. After testing, their model proved to be more efficient on their training set than other types of machine learning methods (support vector machines and NB-based) and was able to identify more positive targets than TargetSpy [120] and miRanda while generating a higher false-positive rate. Using the same training dataset, a multiple instance learning random forest classifier (MIL-RF) called MBSTAR was developed [77]. This model considers potential binding sites as instances and miRNA-mRNA pairs as bags. Thus, a bag can contain several instances. If at least one of the instances is labeled positive, then the bag is labeled as functional. Since the authors of this algorithm deem the secondary structure of the target to be more important than site hybridization, the top features used by MBSTAR are nucleotide patterns in the flanking areas of the potential site and are not seed-related. MBSTAR achieves an accuracy of 78% on a large independent dataset (2nd best is miRanda with 58%). Unfortunately, the authors did not perform a comparison with RFMirTarget, which is the closest related method to MBSTAR. Recently, the authors of TarPmir used data from CLASH (crosslinking, ligation, and sequencing of miRNA-RNA hybrids), a high-throughput experimental method for identifying MTIs, to train an RF-based model for MTI predictions [121]. The advantage of CLASH compared to CLIP-seq experiments is that it provides both the miRNA and the corresponding target sequences. The training dataset was published by Helwak *et al.* in 2013 and contains 18,534 MTIs for 399 miRNAs [57]. Since no other CLASH datasets were available at the time, the performances of this method were tested on three independent PAR-CLIP datasets. Validated MTIs were identified using DIANA-TarBase (v7.0) [109]. Although TarPmir scored better than three other commonly used algorithms, it only achieved 55% recall and 19% precision, leaving much space for improvement. However, since CLASH data include many “nonseed” MTIs, TarPmir can better predict most sites of this type.

####### 3.1.2.3 Support vector machines

**Fig. 5:**
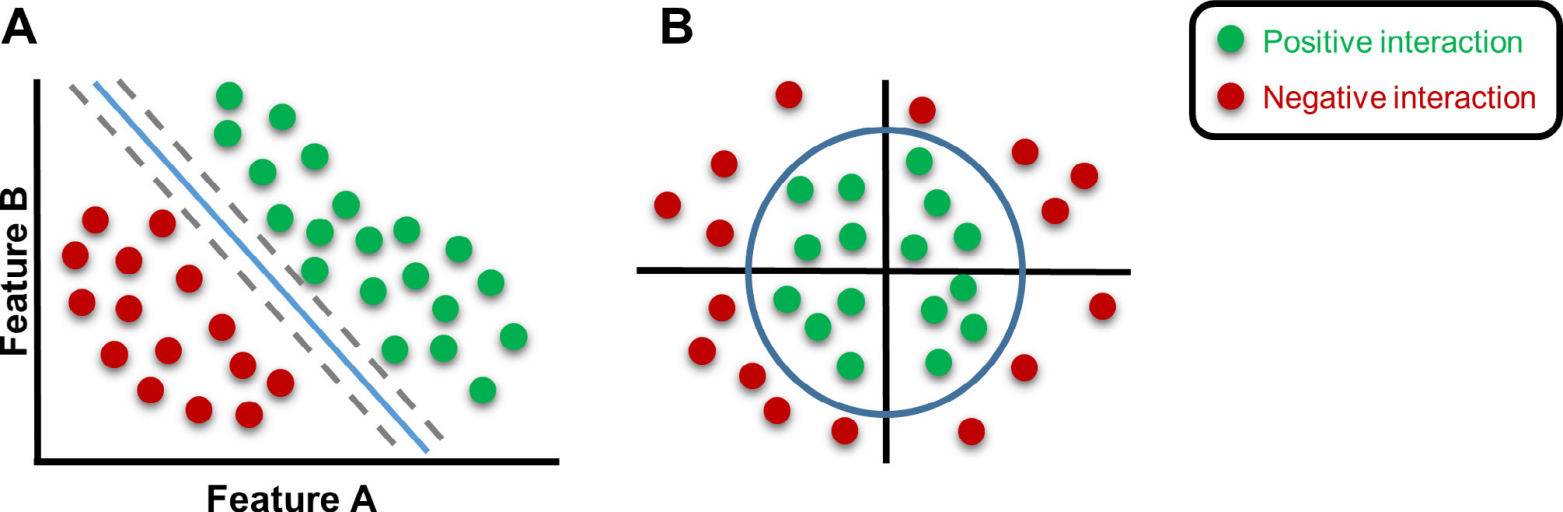
Nonlinear support vector machine (SVM). A) SVM constructs hyperplanes (gray dotted lines) in a multidimensional space (as many as the number of features being used) that separates cases of different class labels. B) Biological data are rarely separable by straight lines, and a transformation is often used to obtain a nonlinear separation model.

Support vector machines (SVMs) are machine learning algorithms generated to identify the best hyperplanes (linear separation between positive and negative data) while maximizing the margin of error. The training data points that are on the margin hyperplanes are called “support vectors”. In the field of biology, however, it is impossible to separate all training data points by a straight line. Thus, some will be located within the margin or on the wrong side of the hyperplane. SVMs are then formulated to soften the impact of these points or use more support vectors. SVMs often use a nonlinear curve to create a decision boundary between data points (Fig. 5) [104]. Most SVMs used for MTI prediction are nonlinear and based on a similarity function called a kernel between pairs of samples (miRNA:mRNA) [89], [91], [92], [99], [122]. MiTarget was one of the first algorithms to implement an SVM to predict MTI and showed equal performances to popular predictors, such as miRanda, TargetScan or RNAhybrid [92]. Interestingly, SVMicrO implemented two SVMs, one for the site and one for UTR-related features [89]. Naturally, the most important feature of the site-SVM is seed-based, although conservation of the 3’ context region of the interaction was the 2nd best ranked feature. The debate over the use of conservation criteria has been quite active in the field of SVM, with some researchers not using it at all and others showing that it is an important parameter [56], [89], [92], [120], [122]. For the UTR-SVM of SVmicrO, predictions result mainly from the number of positive sites in the UTR (the greater the better) and the score of each of these sites (the higher the better) as well as the length of the UTR. At the time of writing this review, SVMicrO showed overall better performance than Pictar, miRanda, mirTarget, TargetScan and PITA; however, this tool has never been updated and is no longer maintained. In the SVM approach MiREE, a hybrid solution is proposed by combining genetic programming for miRNA duplex characteristics (sequence homology and thermodynamics) and a nonlinear SVM for context features [91]. Similar to SVMicrO, its most important features are seed-related. This method obtained a 95% accuracy on human MTI predictions, which is higher than the other methods compared in this review (2nd best is miTarget at greater than 60%). Surprisingly, the Avishkar predictor used a linear SVM model because it has the advantage of being directly interpretable from the weights of each feature and easy to implement [56]. However, as mentioned above, this type of machine learning is expected to perform poorly due to the complexity of MTIs. As a result, even though Avishkar obtained a 98% recall on human MTI, the method showed poor accuracy, with 30% of all predicted targets being misclassified. Interestingly, Li *et al.* proposed improving the performance of miRNA target prediction by searching a second MTI on the whole mRNA sequence after finding one in the 3’-UTR [123]. Thus, they trained an SVM on a two-site search dataset of validated MTIs from miRecords and pSILAC (quantitative proteomics) experiments. When tested on an independent dataset, it showed higher performance than other commonly used methods (PicTar, MirTarget2, miRanda, PITA, TargetSpy, TargetMiner, and TargetScan). To improve both the prediction model and the training dataset, Lu et Leslie created chimiRic, a two-SVM model based on CLASH and AGO-CLIP sequencing data [124]. One SVM uses both data types for duplex prediction, and the other serves for AGO site discrimination (true or not). This strategy has the advantage of training on a large dataset of interacting miR-target duplexes but does not guarantee their functionality. Nevertheless, it shows a superior performance to MIRZA, MirTarget, TargetScan, miRanda and Diana-microT-CDS.

####### 3.1.2.4 Artificial neural networks

**Fig. 6:**
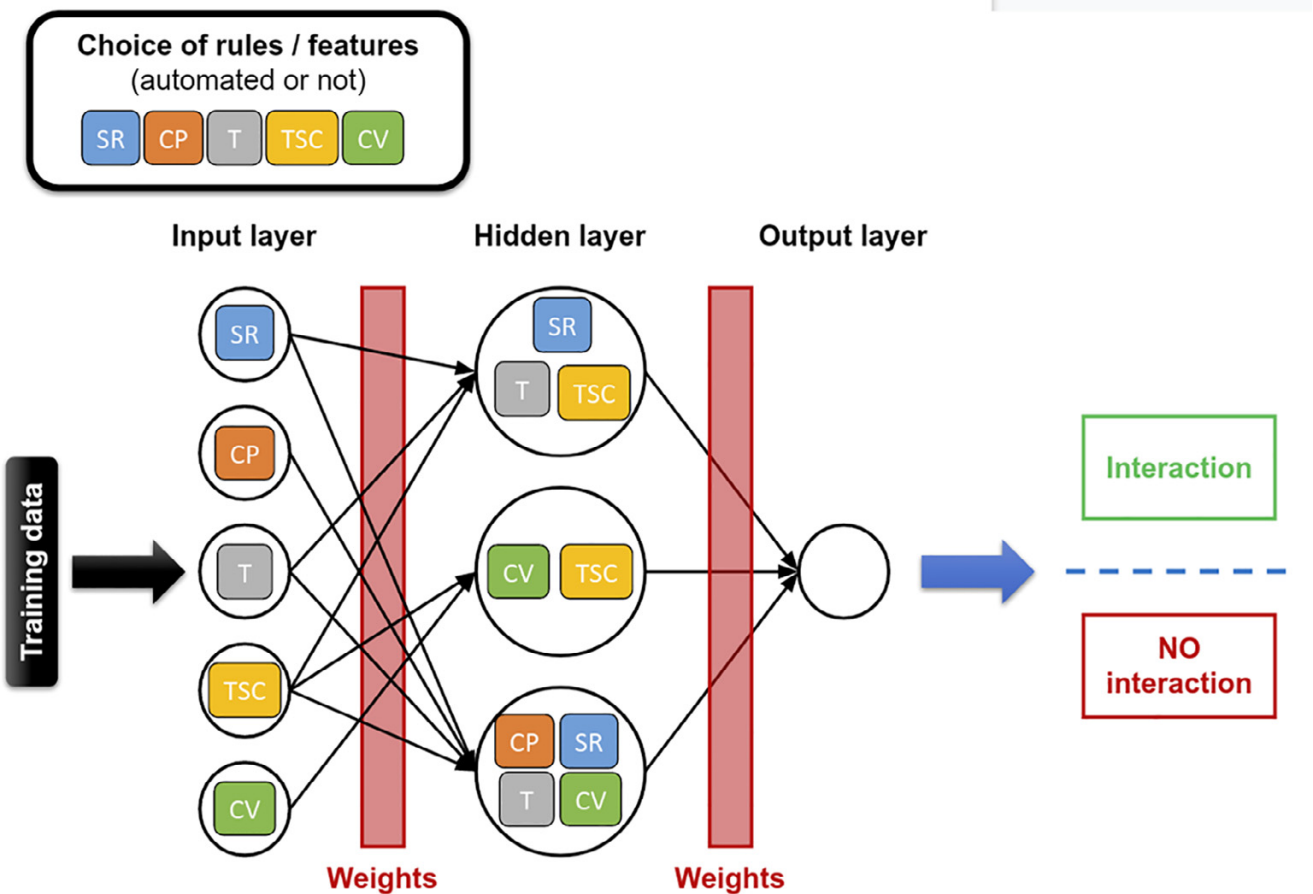
Neural Network. Selected features (here, seed region (SR) pairing, compensation (CP) pairing, thermodynamic (T) pairing, target site context (TSC) pairing and conservation (CV) pairing) are used as input signals in this feedforward partially connected neural network example. Each node decides what to send to the next node following various principles, such as fuzzy logic, genetic algorithms or Bayesian statistics. Weight factors are applied to each edge. Eventually, an output layer will combine all results in one or several nodes (one in this example), thus allowing the classifier to make a decision. The model can change the weights and node ordering to best classify the training data.

Artificial neural networks (ANNs, also called neural networks) have been developed using interconnected neurons in the brain as a model. Features are used as input nodes in this model to feed the “neurons” or working units of the algorithm, which then create new combinations (hidden layers) of these inputs following principles such as fuzzy logic, genetic algorithm or Bayesian statistics, and a prediction is eventually returned. Weight factors are assigned to each neuron to modulate its impact on the predicted result. The model is computed to be adaptive so that weight factors and neuron ordering can change to best suit the training data [125] (Fig. 6). One of the first MTI prediction methods using an ANN was MTar [126]. Unlike most of the current algorithms, which heavily focus on seed region matching, MTar aimed to efficiently identify MTIs regardless of the type of interaction. It first calculates a complementarity score to determine the category into which the site falls: 5’ seed-only, 5’ dominant and 3’ canonical (determined from Betel *et al.* [90]). Three different ANNs were trained depending on the site category. They contain 16 input nodes, 9 neurons in the hidden layer and 1 unit in the output layer. This method produces more than 90% fewer targets for each miRNA compared to conventional methods with 94.5% sensitivity and 90.5% specificity. Using a very similar model to that of MTar, HomoTarget uses a pattern recognition neural network (PRNN) coupled with principal component analysis (PCA) for feature selection [112]. It contains 16 input nodes, 14 neurons in the hidden layer and 2 units in the output layer. Unlike MTar, HomoTarget focuses on the seed region to predict MTIs since it filters sequences based on standard seed rules. HomoTarget was trained on a dataset of 425 examples and showed 99% specificity using cross-validation. These two algorithms quickly achieved high performance values due to the limited number of duplexes in their training and testing datasets. It would be interesting to test them on independent and larger datasets.

####### 3.1.2.5 Training datasets

As mentioned above, a good training dataset needs to have a high number of high-quality examples. The training dataset is a critical aspect of all machine learning methods. A major challenge in creating an MTI dataset is to generate real negative examples. The strategy of creating random nucleotide sequences of varying lengths was tried for a few models but was then quickly disregarded because such sequences often interact with miRNAs, as shown in the signal-to-noise ratio experiments of previous studies [24], [92], [94], [107], [113], [127]. TargetMiner’s authors (who later also created MBSTAR) emphasized this issue [99]. Instead of generating random sequences as negative MTIs, they crossed the predictions of other algorithms (miRanda, TargetScanS, PicTar and DIANA-micro-T) with microarray experiments. If a miRNA and its potential targeted mRNA were both overexpressed in a given tissue, then this pair was retained as a negative example. Using this method, 289 negative MTI were generated. A subset of negative examples was then confirmed on a separate pSILAC dataset [128]. To complete the dataset, 289 experimentally validated positive sites were retrieved from miRecords and TarBase [109], [110], [129]. Using an independent dataset (187 positive and 59 negative pairs), TargetMiner showed 74% accuracy when NBmiRTar and MirTarget2 only had 51% and 46%, respectively, (lower than reported in their original publications), clearly showing the importance of the testing dataset in performance evaluation. Furthermore, they showed that TargetMiner performs better when trained with their negative dataset than with an artificially generated negative set. They confirmed this finding by obtaining similar results with the model of NBmiRTar when repeating the experiment. While validated interactions are most often taken from miRecords or TarBase, some predictors, such as MirTarget2, TargetSpy and Avishkar, were directly trained with positive interactions inferred from microarray or CLIP-seq experiments [56], [120], [122]. The development of high-throughput methods fostered the tendency to include the largest number of interactions regardless of functional testing. Several datasets used by many predictors marked the history of MTI prediction methods, such as those reported by Linsley *et al.* in 2007 (microarray), Selbach *et al.* in 2008 (pSILAC), Chi *et al.* in 2009 (HITS-CLIP) or Hafner *et al.* in 2010 (PAR-CLIP) [53], [128], [130], [131]. As mentioned in the introduction, miRNAs do not necessarily reduce mRNA levels; thus, microarray data insufficient to fully reflect the action of a miRNA. The use of complementary proteomics data is recommended in this case. Moreover, underexpressed mRNA/protein levels measured by high-throughput experiments can be due to indirect effects of miRNA action [132]. Recently, some predictors were trained on CLASH experiments, which identified both AGO-binding miRNAs and target sites on a transcriptome-wide scale. However, some caution must be taken with CLASH data because several issues related to the specificity of the ligation and the functionality and exhaustivity of the captured MTIs remain unsolved [39], [124]. At present, as difficult and expensive as it might be to acquire the data, combining all these technologies (CLIP-seq, CLASH, microarray and pSILAC) seems to represent the best solution for the use of large training datasets.

###### 3.1.3 Commonly used prediction tools

Most if not all prediction algorithms are usually compared to miRanda, Diana-microT-CDS and/or TargetScan because these three heuristic scoring methods have generally be used by biologists to identify MTIs prior to wet-lab experiments. Their popularity is due to their long history, frequent updates and strong adaptation ability to new advances in MTI prediction.

In the direct foot-step method proposed by Stark [85], miRanda (2003) was developed to further identify MTIs in animals. MiRanda uses the ViennaRNA package to calculate the thermodynamic folding energy of interaction and a scoring matrix, and it assigns values for each nucleotide pairing, with the higher scores used for seed matching [88]. Site conservation is also included in the tested features, and the results are ranked according to the conservation score. From 2004 to 2010, miRanda was upgraded to integrate target site context (global, local and at the duplex level), with a final scoring performed by a support vector regression algorithm (mirSVR) based on mRNA expression change [90], [133]. The authors trained mirSVR on a set of nine microRNA transfection experiments performed in HeLa cells by Grimson *et al.* [50]. The score resulting from mirSVR is intended to estimate the efficiency of miRNA action on a given target site and not the probability of regulating this site. With this model, the authors found that the most important features are related to the seed region. The upgrading of mirSVR showed significantly better performances than the previous version of miRanda and seems slightly above TargetScan [90].

Diana-microT is an algorithm published in 2004 that first searches for miRNA-recognition elements (MREs) in the 3′-UTR of a mRNA, including Watson-Crick pairing identification and minimum binding energy calculation using a 38-nt window. A second parameter takes into account the miRNA-associated protein complex, which impacts both the pairing between the miRNA and its target and the site accessibility [134]. In 2009, microT was updated to filter MREs that do not have at least a 7mer in the seed region. The authors also decided to integrate the conservation profiles of MREs using 27 species. Eventually, each considered 3′-UTR is ranked by the weighted sum of the scores of all its identified MREs, and a precision score is calculated by comparing the results with a set of mock miRNAs. An enrichment analysis was also performed with all potential MREs for a given miRNA using the KEGG pathway database. The results are highlighted in the significant pathways that were identified [135]. In 2012, the algorithm was renamed DIANA-microT-CDS because numerous studies have shown that the mRNA coding region can be targeted by a miRNA with a measurable effect on its degradation. Therefore, microT is now used to screen for MREs in this mRNA region, and associated conservation scores are also calculated. Moreover, a dynamic programming algorithm identifies the optimal alignment for the miRNA extended seed sequence (nucleotides 1–9 from the 5′-end of the miRNA) with a 9-nucleotide window on the 3′-UTR or CDS. The prediction method scores the 3′-UTR and CDS region differently and then combines these scores to create the final estimation for the whole mRNA [136]. This last update showed better performance than miRanda and TargetScan at the time of its publication in 2012.

Released as a freely available web tool in 2003 by Bartel’s group, TargetScan first used conservation of miRNAs and mRNA UTRs as a filter and then seed matching (length and frequency), 3′ compensation and folding free energy as prediction features [94], [137]. The algorithm progressively evolved (last version: v7.2, 2018) to take into consideration all analyzable elements of MTIs that were previously described [2], [50], [60], [137], [138], [139]. TargetScan broke down these elements into 14 features using multiple linear regression models (one for each of the four common seed types, off-set 6mer included) trained on microarray datasets published by Garcia *et al.* in 2011 [138]. The resulting models were collectively called the context++ model. When multiple sites are present, individual context++ scores are summed to rank the predicted 3′-UTR. Over time, site conservation has become one of the features of TargetScan and is no longer used as a filter. With a relatively weak contribution to the context++ score, nonconserved targets can even represent the top prediction. After thoroughly analyzing CLIP datasets, the TargetScan authors concluded that “noncanonical sites might exist but have not yet been characterized to the point that they can be used for miRNA target prediction”; therefore, they did not include these sites in their predictions [60]. They also evaluated the use of other more complex types of regression (e.g., linear regression models with interaction terms, lasso/elastic net-regularized regression, multivariate adaptive regression splines, random forest, boosted regression trees, and iterative Bayesian model averaging) but did not find any improvement compared to that of linear regression models [60]. This result is consistent with a similar test performed by Vejnar *et al.* in 2012 [98]. The version of TargetScan described in 2015 showed better performance than 15 other predictors (miRanda and microT included) when tested on the dataset from Linsley *et al.* [131]. With 8 publications describing its content and updates, TargetScan is currently the most widely used MTI prediction tool by the scientific community (more than 1700 citations from PubMed as of November 2020) [102], [140], [141].

#### 3.2 Data combination

Due to the moderate overlap of the results (5–70%) between all previously cited methods [142], investigators often combine the predictions of different tools to obtain mainly true positive MTIs. Several strategies to combine MTI predictions have been proposed as described below.

##### 3.2.1 Union and intersection

Assuming that an interaction predicted by more than one algorithm is more likely to be functional, databases such as miRWalk, miRSystem or miRGator store and compare results predicted by several tools using statistics and/or mRNA/protein expression data [103], [143], [144], [145], [146]. Using such an intersection strategy, Kuhn *et al.* validated the interaction of the human angiotensin II type-1 receptor (hAT1R) with hsa-miR-155 and suggested based on their findings to cross results between at least two MTI predictors before undertaking experimental investigations [147]. Ritchie *et al.*, however, demonstrated that targets resulting from the intersection of two lists of predictions are not more likely to be present in the intersection of two other lists [46]. Therefore, intersecting results do not increase the probability of retaining true positives. Moreover, approaches based on the intersection of predictions may lead to decreased sensitivity by possibly omitting valid interactions, as shown by Sethupathy *et al.* [148]. In fact, Oliveira *et al.* [149] showed that union of the results obtained by several prediction tools was more efficient than their intersection. However, when ranking MTIs is required, this method should not be used since it increases the rate of false positives and therefore decreases the specificity of the predictions, which is the most important aspect for ranking purposes. Nevertheless, these databases have the advantage of giving a wide panel of predictions for a given miRNA, with an edge observed for miRWalk, which has been recently updated [103]. Overall, most users do not have enough understanding of MTI predictions to decide which database to take or remove from the union and intersection strategies to be efficient.

##### 3.2.2 Ensemble methods

Because of the limits of the intersection strategy, the union with a rescoring method has been used to better rank MTIs according to the likelihood of being true. This strategy was first explored by DeConde *et al.* in 2006 using an algorithm that combines ranked lists of miRNA targets from five microarray studies and a reranking of the targets using a statistical test proposed by Tusher *et al.* [150], [151]. The performance of this method compared to other tools was not evaluated. Although this work was performed using experimental data, other methods have used aggregation strategies on MTIs predicted by several commonly used tools. For example, MiRror-Suite gathered predicted and/or validated MTIs from 18 databases and allowed for the analysis of approximately 40,000 genes and 2,500 miRNAs [152]. The aggregation strategy consisted of creating a set of potential targets using several filters (species, miRNA family, cell line, number of databases, etc.) and then calculating the probability of an MTI being functional based on a hypergeometric test. However, the ranking performances of this algorithm were not compared to that of other methods. Alternative strategies were tested, such as ExprTarget, which used a multivariate logistic regression model to combine the scores of 3 databases (miRanda, PicTar and TargetScan) and which clearly outperformed other methods based on aggregation [153]. The good performance of similar combination approaches was also confirmed with a model that aggregated 9 predictive algorithms [154]. Others, such as BCmicrO and ComiR, have used more complex strategies for the combination step with an NB classifier for BCmicrO and an SVM for ComiR [155], [156]. Interestingly, ComiR takes into consideration the expression levels of miRNAs in its rescoring methods. Of note, ComiR was designed to specifically predict the targets of a set of miRNAs and to consider combinatory interactions. As expected, all aggregation methods were able to outperform in terms of MTI ranking and each aggregated database was considered individually. This finding was also confirmed with our aggregation method named miRabel using a very large dataset (982,411 common interactions) [157]. MiRabel uses a statistic R package (RobustRankAgreg) to rescore MTIs according to their ranks in 4 databases (miRanda, PITA, SVMicrO and Target). This recently published method showed better or equal ranking specificity when compared to other (not aggregated) popular prediction tools. The biological relevance of combined miRNA target predictions from multiple prediction algorithms can also be enhanced by prioritizing results based on functional ranking (inferred from Gene Ontology and enrichment analysis) [158].

## 4 Performance evaluation

Since prediction tools are designed for biologists, the ease of use should be a criterion in the overall performance. These tools are usually presented in three different platform types: web service, downloadable programs or R/python packages. The first type is the most commonly used because of its user-friendly features; however, ease of use is generally inversely related to flexibility; thus, this tool offers the least degree of freedom in sequence analysis [140].

Programs that exhibit a greater correlation between their predictions and protein or RNA downregulation are commonly considered state-of-the-art tools [41]; however, this would not be the case if the downregulation was directly due to miRNA transfection, which is far from being certain in high-throughput experiments because the experimental conditions can induce false positives.

A more interesting and widely used evaluation method is the area under the receiver operating characteristic (ROC) curve (AUC), which is now well recognized for its capacity to evaluate the performance of classifiers [159]. It plots the sensitivity or true positive rate (TPR) against specificity or false-positive rate (FPR) with TPR = TP/(TP + FN) and FPR = FP/(FP + TN). An MTI is considered to be a true positive (TP) if it has been predicted and experimentally validated, a true negative (TN) if it has been neither predicted nor validated, a false-positive (FP) if it has been predicted and not validated, and a false negative (FN) if validated but not predicted. TPs are readily available through several databases, but this is unfortunately not the case for tested but not validated interactions. Therefore, in the field of MTI prediction, a nonnegligible part of FPs and TNs are mislabeled, thus creating biases in ROC analyses [154]. To complement the ROC analysis, the precision (TP/(TP + FP)) can be plotted versus the recall (same as TPR), and the AUC can also be used for classifier performance evaluation (PR analysis) [160]. An alternative is to plot the cumulated precision versus the normalized scores (sorted in descending order) [154]. Both methods have the advantage of not taking TN into consideration, which minimizes the number of mislabeled MTIs in the analysis. The problem is not completely solved, however, since the accuracy of these methods still depends on the included FP. The use of both ROC and PR analysis is thus recommended for complete performance evaluation of an MTI prediction tool. Unfortunately, not all published algorithms use the same type of parameters to evaluate the performance, which makes comparisons almost impossible. A common pitfall that has been increasingly avoided is to use the training dataset to evaluate prediction performances. Indeed, using several datasets to truly evaluate the performances of predictors is crucial. To address this issue, several independent reviews have already benchmarked some of the previously presented tools, with some predictors being in all benchmarking papers [102], [146], [161]. Using all the measurements mentioned above and additional measurements, Fan and Kurgan [102] compared 7 target predictors with 4 testing datasets. Although TargetScan and miRmap appeared to be the strongest in this report, a consistent best predictor was not observed across all the possible measurements. Of note, TargetScan performs systematically well in the vast majority of studies comparing MTI prediction algorithms, and it is closely followed by Diana-microT-CDS and miRanda-mirSVR.

These prediction tools are often misused because they do not predict the biological functionality of the interaction between miRNA and mRNA. Indeed, it is unlikely that each predicted miRNA target is sufficiently dose-sensitive to be functionally regulated by miRNAs. Moreover, several studies have shown that some miRNA target prediction software programs are contaminated by high false-positive rates, although this information is rarely emphasized [162], [163]. Thus, some mRNAs can efficiently titrate miRNAs, which may contribute to the conservation of miRNA binding sites for ineffectively repressed targets. Another possible explanation would be that phylogenetically conserved interaction sites are conserved for reasons independent of their interaction with miRNAs, which would lead to the overconservation of seed sequences and thus to an increase in the false-positive rate. A better understanding of MTI prediction will likely improve the performance of these bioinformatics tools.

## 5 Summary and outlook

All miRNA target prediction algorithms use a combination of the sequence, site accessibility and conservation features to identify potential MTIs. However, since the mechanisms of miRNA action are not yet fully understood, predictors still have a high false-positive rate. To improve the accuracy of these tools, different computational methods have been tested. However, none thus far has shown a systematically higher performance regardless of the parameters considered. Surprisingly, empirical methods do not seem to perform better than heuristic methods, suggesting that current training datasets do not efficiently capture all possible MTIs. Additionally, standardization methods are required to compare the algorithms. MTI prediction is challenging, and overcoming the difficulties will require closer coordination between multidisciplinary teams. Overall, 3 predictors, TargetScan, miRanda and Diana-microT, perform well, as reported in benchmarking reviews [102], [146], [161]. Until better algorithms are developed, ensemble methods seem to be the most efficient strategies to obtain an integrated vision of target predictions for a given miRNA. Ultimately, efficient MTI prediction will reduce the time and resources spent validating miRNA targets and therefore increase the ability of molecular biologists to elucidate the role of miRNAs and their targets under physiological and pathological conditions.

## Funding

This work was supported by the Ligue Contre le Cancer de Normandie, Conseil Régional de Normandie, Institut National de la Santé et de la Recherche Médicale (UMRS1239), University of Rouen Normandy and the European Community. Europe has become involved in regional development through the ERDF program. The funding bodies were not involved in the design of the study, the writing of the review or the decision to submit for publication.

## CRediT authorship contribution statement

**Aurélien Quillet:** Conceptualization, Investigation, Writing – original draft. **Youssef Anouar:** Writing – review & editing, Funding acquisition. **Thierry Lecroq:** Investigation, Writing – review & editing. **Christophe Dubessy:** Conceptualization, Writing – review & editing, Supervision, Project administration, Funding acquisition.

## Declaration of Competing Interest

The authors declare that they have no known competing financial interests or personal relationships that could have appeared to influence the work reported in this paper.
